# The life cycle of cyclotides: biosynthesis and turnover in plant cells

**DOI:** 10.1007/s00299-020-02569-1

**Published:** 2020-07-27

**Authors:** Blazej Slazak, Tobias Haugmo, Bogna Badyra, Ulf Göransson

**Affiliations:** 1 Pharmacognosy, Department of Medicinal Chemistry, Biomedical Centre (BMC), Uppsala University, Box 574, 751 23 Uppsala, Sweden; 2 W. Szafer Institute of Botany, Polish Academy of Sciences, 46 Lubicz St., 31-512 Cracow, Poland

**Keywords:** Cyclic peptides, ^15^N labelling, Plant cell suspension culture, *Viola*, Mass spectrometry

## Abstract

**Key message:**

**Turnover rates have implications for understanding cyclotide biology and improving plant cell culture-based production systems.**

**Abstract:**

Cyclotides are a family of polypeptides recognized for a broad spectrum of bioactivities. The cyclic, cystine knot structural motif imparts these peptides with resistance to temperature, chemicals and proteolysis. Cyclotides are found widely distributed across the Violaceae and in five other plant families, where their presumed biological role is host defense. Violets produce mixtures of different cyclotides that vary depending on the organ, tissue or influence of environmental factors. In the present study, we investigated the biosynthesis and turnover of cyclotides in plant cells. *Viola uliginosa* suspension cultures were grown in media where all nitrogen containing salts were replaced with their ^15^N counterparts. This approach combined with LC–MS analysis allowed to separately observe the production of ^15^N-labelled peptides and decomposition of ^14^N cyclotides present in the cells when switching the media. Additionally, we investigated changes in cyclotide content in *V. odorata* germinating seeds. In the suspension cultures, the degradation rates varied for individual cyclotides and the highest was noted for cyO13. Rapid increase in production of ^15^N peptides was observed until day 19 and subsequently, a plateau of production, indicating an equilibrium between biosynthesis and turnover. The developing seedling appeared to consume cyclotides present in the seed endosperm. We show that degradation processes shape the cyclotide pattern present in different tissues and environments. The results indicate that individual cyclotides play different roles—some in defense and others as storage proteins. The turnover of cyclotides should be accounted to improve cell culture production systems.

**Electronic supplementary material:**

The online version of this article (10.1007/s00299-020-02569-1) contains supplementary material, which is available to authorized users.

## Introduction

Cyclotides are a family of plant peptides with unique features. As the name suggests, peptides belonging to this family have a macrocyclic amino acid backbone, which is completed through a peptide bond between the N- and C-termini. The molecule is further stabilized by three disulfide bonds, which create what is referred to as the cyclic cystine knot (CCK) (Craik et al. 1999). Cyclotides are products of genes embedded in the nuclear genome. They are synthesized in the form of precursor proteins that undergo a series of posttranslational modifications—enzymatic cleavage, formation of disulphide bonds and cyclization (Jennings et al. 2001; Nguyen et al. 2014). The matured peptide product is then stored in the vacuole (Conlan et al. 2011; Slazak et al. 2016). The kinetics of the biosynthetic processes have never been studied. Moreover, it is unknown what happens to cyclotides once they are deposited in the vacuole.

Cyclotides have attracted attention due to their biological activities and stability. Multiple studies have indicated different activities including antimicrobial, cytotoxic and hemolytic (Ireland et al. 2006; Herrmann et al. 2008; Pränting et al. 2010; Slazak et al. 2018). Cyclotides demonstrate resistance against physical, chemical and biological factors like temperature, extreme PH and degradation by endo- and exopeptidases (Colgrave and Craik 2004). Yet, there have been no studies investigating cyclotide stability in plant cells.

The structure of cyclotides was first identified in 1995 by Saether et al. via NMR analysis of kalata B1 (kB1), a cyclotide first isolated from *Oldenia affinis* by Lorens Gran 25 years earlier (Gran 1973; Saether et al. 1995). Cyclotides or cyclotide-like sequences (acyclotides) have since been found in Rubiaceae (coffee), Violaceae (violet), Solanaceae (potato), Poaceae (grass), Cucurbitaceae (cucumber) and Fabaceae (pea) plant families (Gran 1973; Göransson et al. 1999; Hernandez et al. 2000; Poth et al. 2011, 2012; Nguyen et al. 2013; Burman et al. 2015). Species from the *Violaceae* are a source of large numbers of different cyclotides. It is the only plant family that so far has shown cyclotides in every investigated species (Burman et al. 2015).

The plants producing cyclotides accumulate high quantities of these peptides with concentrations of up to 1.5 g per 1 kg of plant material have been demonstrated (Göransson et al. 1999; Ireland et al. 2006). The investment of resources in peptide production suggests that they play an important role in host plant biology. Considering the anti-insect larvae activities of kB1, it has been hypothesized that cyclotides are host defense peptides (Jennings et al. 2001). Not only one but multiple, even dozens of different peptides are produced by plants (Burman et al. 2015). Complex mixtures—suites of cyclotides—are not only species-specific but also specific to individual plant organs and even tissues (Gilding et al. 2016; Slazak et al. 2018). The production of cyclotides is influenced by the environment. Different quantities of particular peptides are found in the same species, depending on the location or season (Trabi et al. 2004; Simonsen et al. 2005). This is apparently caused by changes in cyclotide gene expression levels but decomposition rates may also contribute in shaping the final suite of peptides. It can be also disputed if the nutrients allocated into cyclotides can be retrieved when resources are scarce, like in case of storage proteins (Slazak et al. 2015).

Cyclotides are a promising compound class with both medical and agricultural applications, such as crop protection. However, obtaining these peptides through chemical synthesis is expensive and, in some cases, difficult; therefore, in most cases, the peptides are still extracted from plant material. To increase yields, in vitro plant cell culture systems were developed for some cyclotide-producing species (Dörnenburg et al. 2008; Slazak et al. 2015; Narayani et al. 2018). The kinetics of cyclotide biosynthesis and decomposition in plant cells are very important for optimization of such production systems. It pinpoints the moment for culture harvesting, optimal biomass production and its cyclotide content.

One way to gain insight into biosynthetic processes and protein turnover is the application of ^15^N isotope labelling. Plants cannot assimilate atmospheric nitrogen; therefore, this macronutrient is obtained by the in vitro culture, entirely from the growth media. In Murashige and Skoog (MS) medium, nitrogen is supplied in the form of two salts: ammonium nitrate and potassium nitrate (Murashige and Skoog 1962). The replacement of these two salts with ones containing ^15^N isotopes results in isotope labelling of amino acids and, in consequence, peptides and proteins (Kim et al. 2005; Schaff et al. 2008). The labelling allows to distinguish newly synthesized peptides from those present in the cell that are broken down over the time of culture.

The current work investigated the biosynthesis and turnover of cyclotides in *V. uliginosa* cell suspension cultures using ^15^N-labeling and in germinating *V. odorata* seeds.

## Experimental procedures

### Growth media preparation and culture conditions

Medium used for cell suspensions was based on Murashige and Skoog 1962 (MS) and supplemented with 2 mgl^−1^ kinetin (KIN) and 2 mgl^−1^ 2,4-dichlorophenoxyacetic acid (2,4-D). For isotopic medium, the macronutrients of MS were added to a MS basal salt micronutrient solution, with ammonium nitrate and potassium nitrate being substituted by their isotopic counterparts. All media and components were obtained from Sigma-Aldrich (Merck Group, St Louis MO). The ^15^N-salts were obtained from Cambridge Isotope Laboratories, Inc., USA. All ingredients and their final concentrations are reviewed in Table 1. All solids were stirred till fully dissolved in milliQ-water. After preparation, the media pH was adjusted with HCl and NaOH to between 5.7 and 5.8 and autoclaved. All cultures were maintained at 23 °C and 18 h/6 h (day/night) photoperiod in a culture cabinet equipped with luminescent light source.

**Table 1:** Components and their final concentration in MS growth medium

Component	Final concentration
NH_4_NO_3_*	1650 mg/l
CaCL_2_	440 mg/l
MgSO_4_	370 mg/l
KHPO_4_	170 mg/l
KNO_3_*	1900 mg /l
Sucrose	30 g/l
Hormones (KIN resp. 2,4 D)	2 mg/l
MS basal salt micronutrient solution	100 ml/l

^*^Salts replaced with ^15^N counterparts in the isotope enrichment media

### Suspension cultures

*V. uliginosa* suspension cultures were initiated and maintained as described earlier (Slazak et al. 2015). 3-week-old callus tissue obtained from leaf fragments cultured on MS media supplemented with 2 mgl^−1^ KIN and 2 mgl^−1^ 2,4-D solidified with agar was transferred to liquid media of the same composition and placed on the rotary shaker. After three weeks of shaking, the callus tissue was drained out and the cell suspension was sub-cultured every two weeks for 4 months prior to the experiments.

### Preparation of suspensions with ^15^N media

Under aseptic conditions, a 2-week-old *V. uliginosa* cell suspension was filtered *in vacuo*. The cells were weighed in a beaker and re-suspended in ^15^N-enriched MS media, reaching concentrations of 6 g of cells per 100 ml of medium. For each of the three replicates per every time point, 15 ml of the suspension was transferred into a separate 50 ml E-flask. The flasks were placed on a shaking table at 130 RPM in the culture cabinet. Cultures for each of the three replicates per every time point were maintained in separate E-flasks (15 flasks in total). The total cyclotide content in the suspensions was compared, including peptides released into the medium. At each time point, for every replicate, the whole suspension culture (15 ml, media and cells) was moved into a 50 ml Falcon tube and freeze-dried. Cultures were collected after 5, 12, 15, 19 and 22 days (T1–T5, respectively).

Additional samples were prepared for the assessment of long-term culture. The suspensions were prepared in a similar way as described above: 4 g of cells was cultured in 100 ml of media. After 48 days, 10 ml of suspension was taken and freeze-dried.

### *V. odorata* seedlings sample preparation

*V. odorata* seeds were obtained from commercial provider (Vilmorin, Poland). Seeds geminated only after stratification and removal of seed coating. Following stratification in 4 °C for at least 2 months, the seeds were surface-sterilized with 70% ethanol for 1 min and then 15 min in 1:1 mixture of commercial bleach:water, and subsequently rinsed with distilled water. Sterilized seeds were placed in a beaker containing wet paper and stored in 4 °C for 7 days to soften the seed coating. Subsequently, the seed coating was removed and the seeds were put on half-strength MS media solidified with 7 g/l agar (Sigma-Aldrich). Seeds were germinated in the culture chamber and 3 different stages of seedling development were collected and freeze-dried: the seed, germinated seedling with endosperm, seedlings that used the whole endosperm.

### LC–MS analysis

After switching to the ^15^N media, the ^14^N cyclotides are diluted in the growing culture as the biomass increases but levels of ^14^N peptides does not. The method for LC–MS analysis was developed to counteract changes in the biomass and the medium during the experiment. Each flask was treated as a separate sample that, after freeze-drying, was extracted in a fixed volume of 3 ml of 30% acetonitrile, (ACN) 0.1% formic acid (FA) in water and placed on a shaker for stirring (24 h, 125 rpm). Following extraction, samples were centrifuged and 0.5 ml of supernatant was acquired, diluted to 10% ACN, 0.1% FA in water with aq. 0.1% FA. *V. odorata* seedling samples were ground to powder and then extracted with 30% ACN, 0.1% FA (200 µl/mg of dry sample) using a TissueLyser (Qiagen, Germantown, MD), 2.5 min, 25 Hz. Equal volume of all the samples was injected into nanoAcquity UPLC/QTof Micro (Waters, Milford, MA) equipped with Acquity UPLC® M-Class Peptide BEH C18 column (75 μm × 150 mm, 130 Å, 1.7 μm; Waters). The peptides were separated using a linear gradient of 1% to 90% ACN, 0.1% FA over 50 min and analyzed in positive ionization using a mass window of 300 to 2500 m/z. Cyclotides produced by the *V. uliginosa* suspension cultures were identified before (Slazak et al. 2015). The monoisotopic masses of peptides expected to be produced by the suspensions together with m/z values of their 3 + ions and monoisotopic m/z of the fully ^15^N-labelled peptides are listed in Table 2. All peptides except one, named after its experimentally determined monoisotopic mass, 3097, have known sequence. The peptide 3097 was included in the analysis because it is a major cyclotide produced by the culture (Slazak et al. 2015). Peptides in *V. odorata* seedling were identified as cyclotides by their late elution profile and molecular mass of 2800–3500 Da (Burman et al. 2015; Slazak et al. 2018). All experiments were done in three independent biological replicates and the mean intensities of monoisotopic peaks for different cyclotides were compared between time points. Means ± standard deviation (SD) of three independent experiments was compared using one-way analysis of variance (ANOVA) with Tukey post test in GraphPad Prism software (GraphPad Software, CA, US). Differences with a *p* < 0.05 were considered statistically significant.

**Table 2:** Characteristics of cyclotides expected to be found in the *V. uliginosa* suspension

	Sequence	Monoisotopic mass	m/z 3 +	Total N atoms	^15^N m/z + 3
cyO13	GIPCGESCVWIPCISAAIGCSCKSKVCYRN	3122.37	1041.80	37	1054.13
cyO2	GIPCGESCVWIPCISSAIGCSCKSKVCYRN	3138.36	1047.13	37	1059.45
mram 8	GIPCGESCVFIPCLTSAIGCSCKSKVCYRN	3113.37	1038.79	37	1051.12
cyO3	GIPCGESCVWIPCLTSAIGCSCKSKVCYRN	3152.38	1051.80	37	1064.13
3097*	n/a	3096.70	1033.24	n/a	n/a

^*^Unknown peptide named after observed monoisotopic mass

## Results

### ^15^N isotope incorporation into cyclotides

**Fig. 1:**
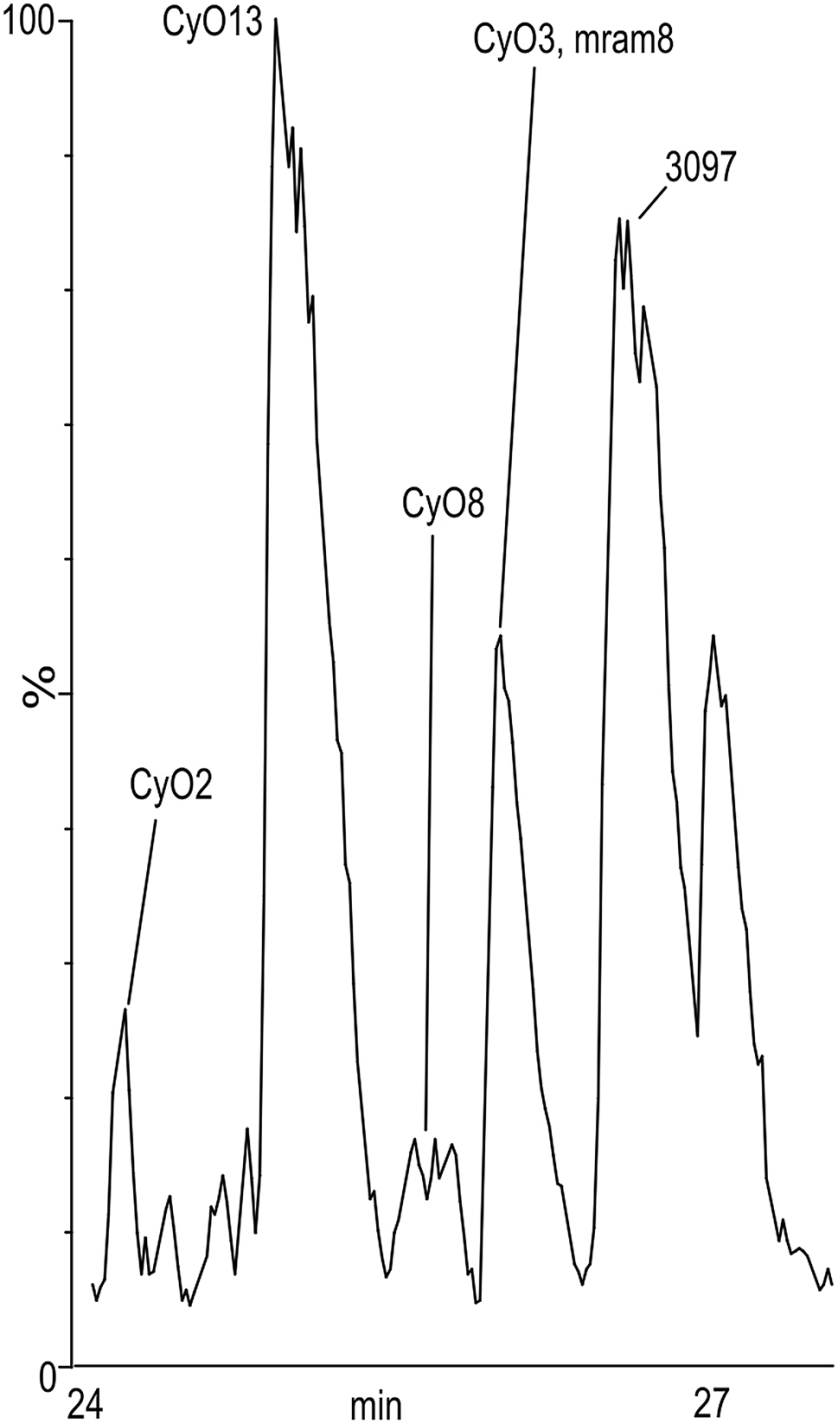
LC–MS chromatogram of extract from *V. uliginosa* cell suspensions cultured in ^15^N media for 12 days

**Fig. 2:**
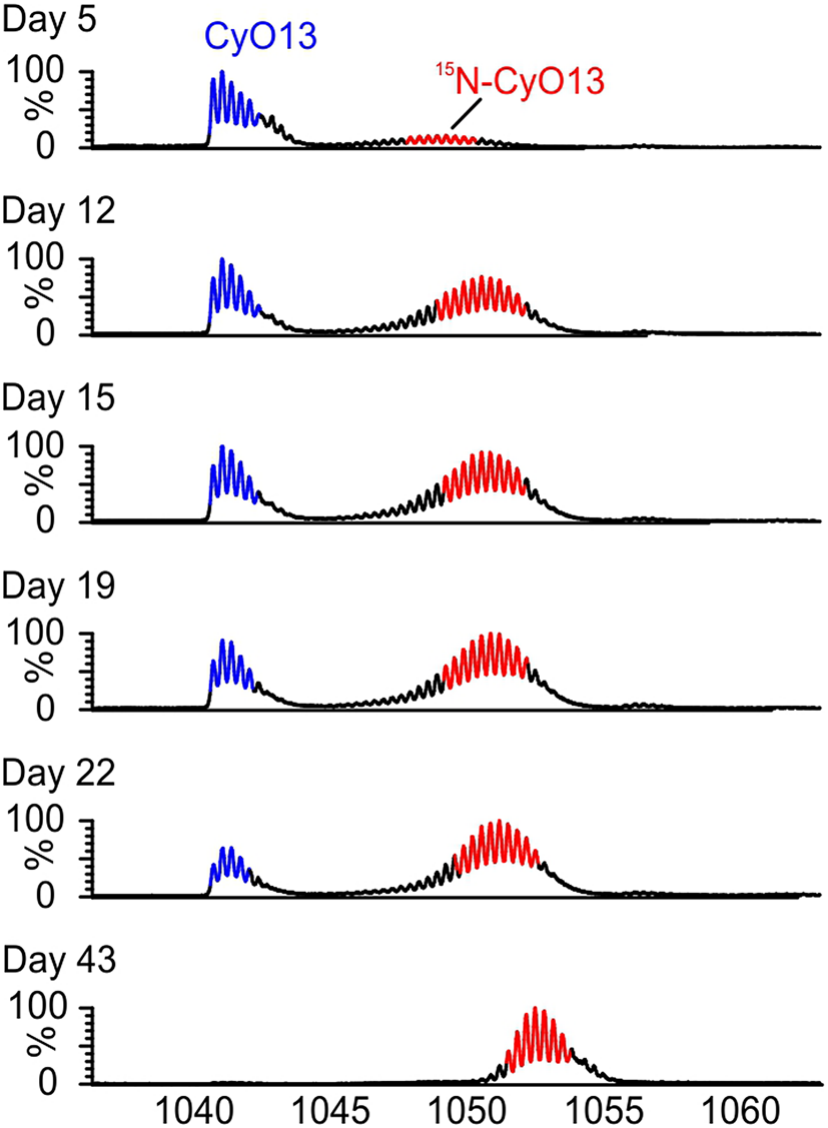
Mass spectra of the cyO13 peak in LC–MS chromatogram of an extract of suspension grown in ^15^N media. The ^15^N-labelled peptides with higher molecular mass and characteristic isotopic pattern (red) emerge aside to ^14^N peptides (blue). With time of culture, the mass shifts towards the mass of the fully labelled ^15^N peptide and the ^14^Ncyclotides disappear. These spectra are representative for other cyclotides present in the extracts (color figure online)

Initially, we investigated the cyclotide biosynthesis in *V. uliginosa* cell suspension cultures using ^15^N growth media allowing for introduction of ^15^N isotopes into the peptides. LC–MS analysis of the extracts showed that the main cyclotides produced were cyO13 and 3097; small quantities of cyO3, cyO8 and mram8 were also detected (Fig. 1). Peptides with a higher mass and a characteristic isotopic pattern resulting from incomplete replacement of nitrogen atoms with a heavier isotope appeared in extracts from suspensions grown in ^15^ N media (Fig. 2). In parallel, the intensities of ions corresponding to monoisotopic ^14^N cyclotides decreased, demonstrating that cyclotides are also being degraded. With progression of time of culture, cyclotides were saturated with ^15^N; the mass of the peptides shifted towards the mass of a fully labelled peptide (all ^14^N atoms replaced with ^15^N). The changes in mass spectra at different times of culture for other peptides are shown in Supplement 1. The ^14^N peptides disappeared completely leaving only the fully ^15^N-labelled in 43 days of suspensions cultured (Fig. 2).

### Turnover rates vary for different peptides

**Fig. 3:**
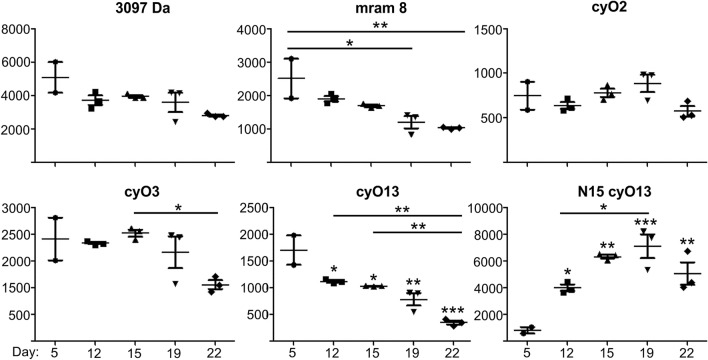
The rates of turnover and biosynthesis of different peptides produced in *V. uliginosa* suspension cultures indicated by intensities of + 3 monoisotopic peaks. The signal drops for various peptides at different pace. The levels of ^15^N-labelled cyO13 increase quickly but reaches a plateau at day 22. The plots represent means ± standard deviation (SD) of three independent experiments. *, **, *** statistical significance level compared to T1 of *p* < 0.05, *p* < 0.01, *p* < 0.001 respectively. Asterisks above bars indicate the level of significance between respective timepoints

The changes in relative quantities of ^14^N cyclotides indicated by monoisotopic + 3 ions peak intensities were analyzed (Fig. 3). Gradual drop of signal was observed for cyO13 and mram8, implying degradation of the peptides over time. The highest rates of degradation were noted for the most abundant cyclotide produced by the suspension, cyO13. The relative quantity of this peptide fell by approximately 80% in 22 days. The levels of cyO2 remained stable and signal for the cyclotide denoted with molecular mass of 3097 dropped gradually until T5 (22 days), but the differences were not statistically significant. The signal intensity for cyO3 was stable but the degradation rates increased in later time points, between T3 and T5 (Fig. 3).

### Cyclotide biosynthesis rates depend on the age of culture

We investigated the biosynthesis of ^15^N cyO13 indicated by changes in intensity of + 3 ions of fully labelled (all 37 ^14^N replaced with ^15^N) peptide (Fig. 3). The amount of fully labelled ^15^N increased very rapidly until day 15. Then, the biosynthesis appeared to slow down between day 15 and day 19. The relative quantity of ^15^N cyO13 in the suspensions sized to grow in T5 (Fig. 3).

### Some cyclotides present in the seed endosperm are digested by the developing *V. odorata* seedling

**Fig. 4:**
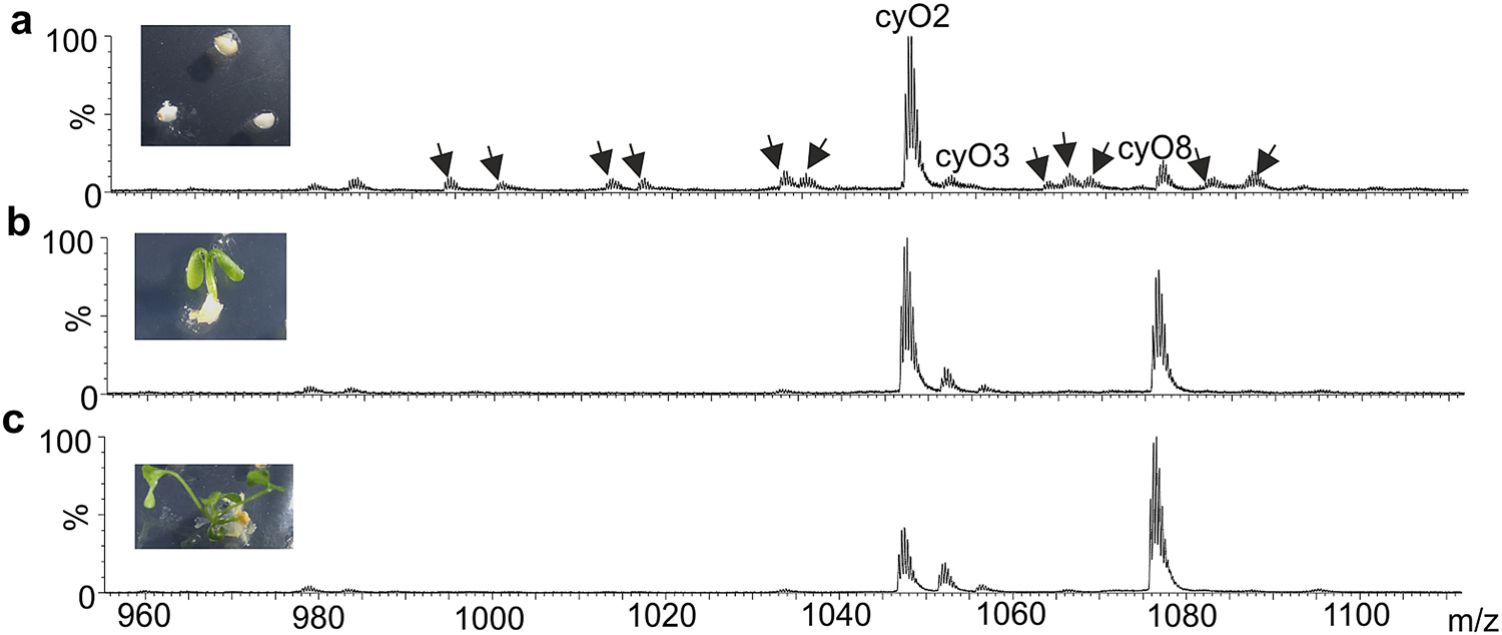
MS spectra of the cyclotide region of the LC–MS analysis of extracts from *V. odorata* seedling on different stages of development: the seed with removed coating (**a**), germinating seedling with remains of the endosperm (**b**) and fully developed seedling (**c**). The peaks corresponding to cyclotides that disappear in the course of seed germination are marked with arrowheads

We observed changes in cyclotide pattern in extracts from *V. odorata* seeds at different stages of germination. Eleven peaks corresponding to different peptides present in the seed disappeared from the average mass spectra of extracts made from fully developed seedlings (Fig. 4). During the germination process, the seedlings digested the seed endosperm (Fig. 4).

## Discussion

The present study shows the application of ^15^N labeling in plant suspension culture to investigate cyclotide turnover and biosynthesis in plant cells.

In the present study, we used mass spectrometry for analysis and ion intensities as a relative measure of quantity. Usually, when mass spectrometry is used quantitatively, especially for absolute quantity measurements, an internal standard is applied to counteract systematic and statistical errors that may occur during analysis. The standards are required to be very similar or identical to the analyte, their production usually involves isotope labeling (Zhu and Desiderio 1996). However, the present study already involved isotope labeling. Standards used in such a study would need to be labeled with isotopes other than ^15^N, carefully selected for each of the investigated peptides. Production of such labeled peptides could be not feasible and introducing them could complicate the spectra and the analysis. Instead, we focused on relative abundance measurements. The use of LC–MS allowed us to separate the peptides from other compounds present in the extract, which might be a source of ion suppression or other systematic errors. This approach allowed easy, conclusive comparisons for multiple peptides. The observed effects were strong enough to reach statistical significance.

In an earlier study by Mylne and Craik 2008, whole-plant ^15^N labelling was used to produce ^15^N cyclotides in *Oldenlandia affinis*. Plants were grown from seeds on media with nitrogen source salts replaced with their ^15^N counterparts. The labeled peptides were then used to aid NMR structure determination of cyclotides found in the plant (Mylne and Craik 2008). Similar to the present study, Mylne and Craik, 2008 did not observe any differences between ^14^N- and ^15^N-containing media in the cyclotides being produced. The same cyclotides were seen in the current study as in the previous work, where the *V. uliginosa* suspension cultures were first initiated (Slazak et al. 2015). In Mylne and Craik, 2008, the plants were harvested after 2 months of culture on ^15^N medium and, when extracted, the peptides were found fully labelled, and all nitrogen atoms were replaced. We sampled our suspensions at different time points and observed a gradual saturation of the peptides. This is because at a given time point, a pool of unlabelled amino acids still exists in the cell; therefore, peptides are made up of a mixture of ^14^N and ^15^N. We observed full labelling of the peptides after 43 days of culture. The present study was not aimed at production of labelled peptides but, in the future, this system can be amended for rapid, efficient cyclotide labelling. In Slazak et al., 2015, it was possible to obtain rapid growth of the culture with approximately 3 days, doubling time and yields > 3 mg of cyO13/g of dry weight. The advantage of labelled peptides is they can be utilised in NMR-based peptide structure determination, in studies on plant physiology or pharmacokinetics and as internal standards in mass spectrometry-based quantitation.

The current work demonstrates that cyclotides are degraded in plant cells. In many previous studies, these peptides are referred to because of their great resistance to degradation by proteolytic enzymes, after ingestion by animals as well as in the environment (Colgrave and Craik 2004; Gunasekera et al. 2008; Chan et al. 2011; Aboye and Camarero 2012). The first cyclotide discovered, kB1 was isolated as the active, uterotonic component of *Oldenlandia affinis* extracts. The indigenous population used decoction of the plant administered orally, to facilitate child birth. Oral bioavailability provides an example of the exceptional stability of cyclotides, to have its effect, kB1 apparently resists boiling and degradation in human digestive system (Gran 1973). Systemic distribution after oral administration, as a consequence of resistance of the molecule, was shown also for kB1 synthetic mutant peptide, [T20K]kB1, in mice (Thell et al. 2016). Recently, we showed that some cyclotides from *V. odorata* resist degradation in the presence of plant pathogenic fungus (Slazak et al. 2018).

The present study indicates that the degradation rates vary, depending on the amino acid sequence of a particular cyclotide. This is probably caused by varying stability and resistance to proteolysis of different sequences. However, the differences in amino acid sequences of many of the cyclotides produced by *V. uliginosa* cultures are very small. For example, cyO2 is different from cyO13 in only one position (Slazak et al. 2015). It indicates that regulation of the turnover rates of particular cyclotides can be very specific. It can also be that each cyclotide evolved for a specific target. In such a case, the peptides that are degraded more slowly could be more valuable, whereas the ones that are degraded quickly, either not needed or more toxic for the plant. In the present work, the cyclotides (e.g. cyO3) appeared to be degraded much faster in the later time points. The drop of relative quantity lagged at time points directly following transfer to ^15^N media. This might have been caused by the presence of ^14^N amino acids in the cells upon the transfer. At the beginning of the culture cyclotides may be still produced from these ^14^N amino acids. If the production rates match the degradation, the effect will be perceived as a lag in degradation. When the ^15^N amino acids are synthesized and subsequently incorporated into the cyclotides, the biosynthesis and turnover finally separate and it is indicated by the acceleration of degradation. Mixtures of cyclotides produced by a plant vary in its different organs and tissues, they are also subjected to seasonal changes and influenced by the environment (Trabi et al. 2004; Trabi and Craik 2004; Simonsen et al. 2005; Ireland et al. 2006; Gruber et al. 2008; Slazak et al. 2015, 2018). The present results indicate that different suites of cyclotides are shaped not only through regulation of gene expression but also by turnover processes. Therefore, a very stable peptide does not require high expression levels to build up abundance over time. This is very important for studies aiming at determination of cyclotides’ biological role in plant defense mechanisms. So far, the issue of the response of the plants to stress in terms of cyclotide production was touched only in a handful of studies. In Mylne et al. 2010, the expression levels of a few genes were compared between plants treated with different stress factors and control. The authors found no response and concluded that cyclotides do not play part in stress response and are a part of the innate defense system (Mylne et al. 2010). However, this study investigated only few cyclotide genes and comprised no biological replicates (Mylne et al. 2010). Moreover, it should be kept in mind that cyclotides are ribosomally synthesized and post-translationally modified peptides, and the production is also directly influenced by translational processes (Jennings et al. 2001). Based on the results of the present study, it seems that the best way to investigate the response to stress should include the peptide level. In a study by Dörnenburg 2010, the biosynthesis of cyclotides in *O. affinis* cultures treated with a biotic elicitor chitosan was investigated. This work showed the response on the peptide level and higher quantities of cyclotides per g of dry biomass were found in the treated cultures compared to controls (Dörnenburg 2010).

Previously, we reported that the *V. uliginosa* suspension culture gives the highest cyclotide yields per g of dry biomass during the exponential growth phase. The yields were much smaller at the plateau phase. This was attributed to cell death caused by nutrient deficiency and release of the peptides to the medium (Slazak et al. 2015). In the current study, we monitored the biosynthesis of cyO13 in the whole culture, including the peptides released to the medium. The relative quantity of peptides increased rapidly but reached a plateau at the end of the studied period of time. In light of this result, it seems that cyclotides can be synthesized quickly upon stress, when nutrients are available. In the following phases, the synthesis reaches an equilibrium with turnover resulting in the observed plateau. Even later, when the nutrients are scarce, the peptides are broken down quicker, then biosynthesized. As a result of this, the signal indicating relative quantity decreases. So far, there has been several successful attempts to develop in vitro culture cyclotide production platforms for different species: *V. odorata*, *V. uliginosa* and *O. affinis* (Seydel and Dörnenburg 2006; Dörnenburg et al. 2008; Dörnenburg 2010; Slazak et al. 2015; Narayani et al. 2017). The understanding of the cyclotide biosynthesis and turnover in such systems is essential for optimization of their effectiveness in terms of biomass and yields.

Plants produce very high amounts of cyclotides, making them an expensive investment of resources, especially nitrogen (Gran 1973; Ireland et al. 2006). The results of the present study indicate that cyclotides can be broken down in the plant and the resources reused. As an example of a situation where these peptides can be used as storage material, we investigated a developing *V. odorata* seedling. We observed the disappearance of peptides that are likely cyclotides described before as endosperm-specific (Slazak et al. 2018), in the time when the seedling used all the endosperm for development.

## Conclusion

The present study shows the biosynthesis and turnover of cyclotides in plant cells. These processes appear to be involved in shaping cyclotide suites present in particular plant organs and as a response to the environment. Cyclotides, depending on the type, may play multiple roles in their host plants, and some of them may be produced for host defense, whereas others as storage material. The findings of the study will aid the optimization of cyclotide production systems.

## Electronic supplementary material

Below is the link to the electronic supplementary material.

Supplementary file1 (JPG 3642 kb)
