# Supplementary figures and images for: The life cycle of cyclotides: biosynthesis and turnover in plant cells
Source: Plant Cell Rep. 2020 Jul 27;39(10):1359–67. doi: 10.1007/s00299-020-02569-1 (PMC7497429; doi:10.1007/s00299-020-02569-1)

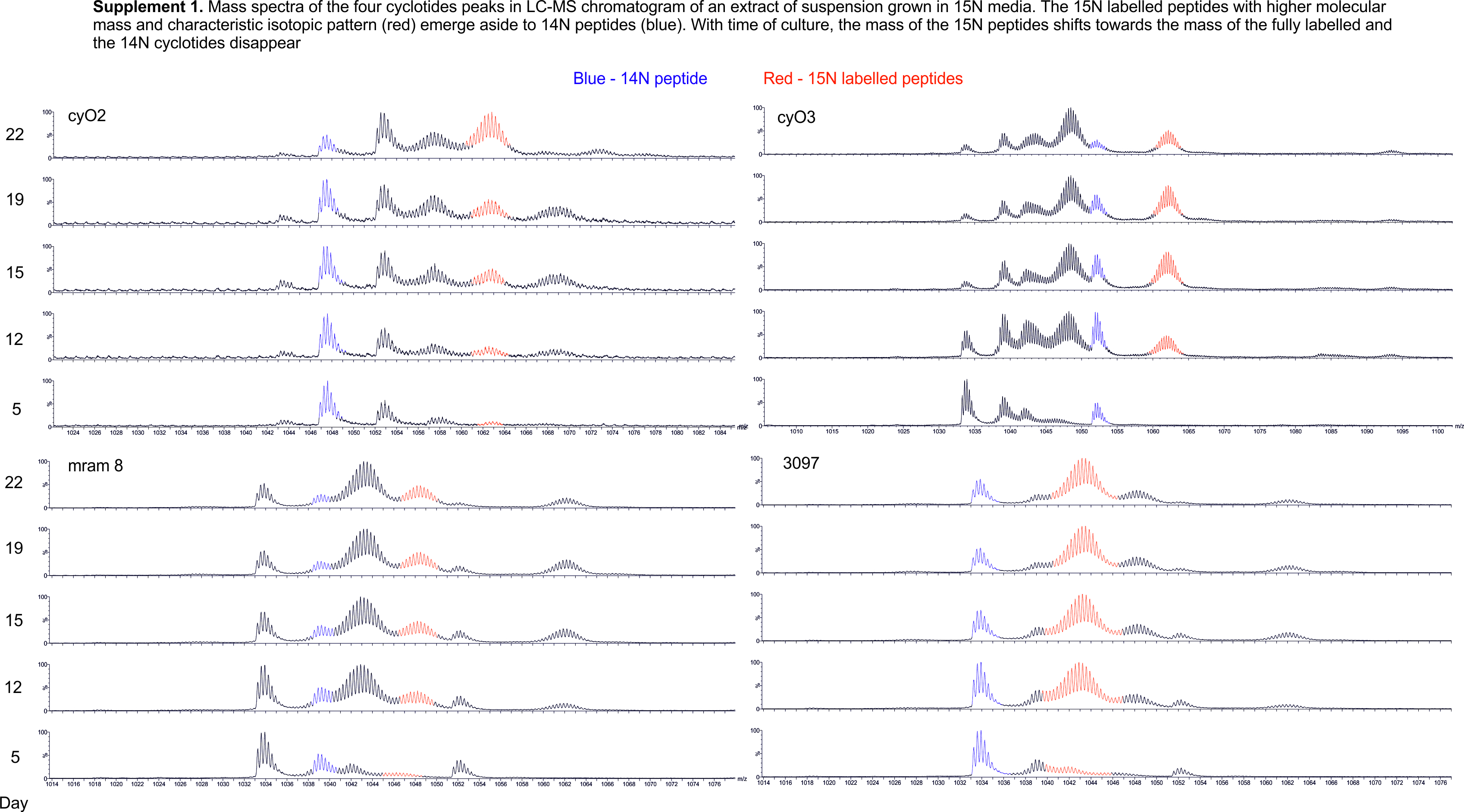

Supplement: Supplementary file 1 — Supplementary file1 (JPG 3642 kb) [file 299_2020_2569_MOESM1_ESM.jpg]
